# *Tri11*, *tri3*, and *tri4* genes are required for trichodermin biosynthesis of *Trichoderma brevicompactum*

**DOI:** 10.1186/s13568-018-0585-4

**Published:** 2018-04-17

**Authors:** Xuping Shentu, Jiayi Yao, Xiaofeng Yuan, Linmao He, Fan Sun, Kozo Ochi, Xiaoping Yu

**Affiliations:** 1 Zhejiang Provincial Key Laboratory of Biometrology and Inspection & Quarantine, College of Life Sciences, China Jiliang University, Hangzhou, 310018 Zhejiang China; 2 Department of Life Science, Hiroshima Institute of Technology, Hiroshima, Japan

**Keywords:** *Trichoderma brevicompactum*, *tri3*, *tri4*, *tri11*, Gene deletion, Trichodermin

## Abstract

*Trichoderma brevicompactum* and *T. arundinaceum* both can synthesize trichodermin with strong antifungal activity and high biotechnological value. The two *Trichoderma* species have a *tri* cluster, which includes seven genes (*tri14*, *tri12*, *tri11*, *tri10*, *tri3*, *tri4*, and *tri6*) that encode transport and regulatory enzymes required for the biosynthesis of trichodermin. Here, we isolated *T. brevicompactum* 0248 transformants with disrupted *tri11*, *tri4*, or *tri3* gene. We also described the effect of *tri11*, *tri3*, or *tri4* deletion on the expression of other genes in the *tri* cluster. Targeted *Δtri3* knockout mutant exhibited a sharp decline in the production of trichodermin, and trichodermol, which is a substrate for trichodermin production, accumulated. Thus, the results demonstrated that *tri3* was responsible for the biosynthesis of trichodermin, and the *tri3* gene-encoded enzyme catalyzed the acetylation reaction of the hydroxy group at C-4 of the trichodermin skeleton. In addition, *tri4* and *tri11* deletion mutants were generated to evaluate the roles of *tri4* and *tri11* in trichodermin biosynthesis, respectively. Deletion mutant strain *Δtri4* or *Δtri11* did not produce trichodermin in *T. brevicompactum*, indicating that *tri4* and *tri11* are essential for trichodermin biosynthesis. This is the first to report the function of *tri3*, *tri4* and *tri11* in *T. brevicompactum*, although the role of *tri4* and *tri11* has already been described for *T. arundinaceum* by Cardoza et al. (Appl Environ Microbiol 77:4867–4877, 2011).

**Electronic supplementary material:**

The online version of this article (10.1186/s13568-018-0585-4) contains supplementary material, which is available to authorized users.

## Introduction

*Trichoderma* species are well-known biological control agents of diseases in numerous crops, and these species produce many antifungal compounds and cell-wall-degrading enzymes (Harman 2006; Malmierca et al. 2012; Tijerino et al. 2011a, b). The biocontrol activities of *Trichoderma* spp. against phytopathogenic fungi generally include antibiosis, parasitism, and competition for space and nutrients (Harman 2006). Antibiotic molecules synthesized by *Trichoderma* are low-molecular-weight and volatile metabolites as well as high-molecular weight polar metabolites. The former type includes simple aromatic compounds, polyketides, volatile terpenes, and isocyanide metabolites, while the latter includes peptaibols and diketopiperazine-like gliotoxin and gliovirin compounds (Reino et al. 2008; Szekeres et al. 2005; Tijerino et al. 2011a). Trichothecenes belong to a large group of terpenoid-derived secondary metabolites and are mainly synthesized by *Fusarium* and other fungal genera, such as *Trichoderma*, *Myrothecium*, *Spicellum*, *Stachybotrys*, and *Trichothecium* (Shentu et al. 2014a; Wilkins et al. 2003). Terpenes are derived from the repetitive fusion of branched five-carbon units based on an isopentane skeleton, and most of the chemical intermediates in their biosynthetic pathway have been identified (Tijerino et al. 2011b). The trichothecene biosynthetic pathway in *Fusarium* has been documented and extensively reviewed (Kimura et al. 2007). However, the genes involved in trichothecene biosynthesis in the other genera remain unknown.

Trichodermin and harzianum A (HA), which are synthesized by *T. brevicompactum* and *T. arundinaceum*, belong to terpenes and have similar structures except for the side chain group at C-4 (an acetyl group and an octa-2,4,6-trienedioic acid, respectively) (Malmierca et al. 2012). *T. arundinaceum* has been used as a model to study the beneficial effect of trichothecenes on the *Trichoderma* biocontrol activity and modulation of plant defense responses of this fungus (Malmierca et al. 2012). Furthermore, bioactivity assays have shown that trichodermin synthesized by *T. brevicompactum* exhibits stronger antifungal activity against *Saccharomyces cerevisiae*, *Kluyveromyces marxianus*, *Candida albicans*, *Aspergillus fumigatus*, *Botrytis cinereal*, and *Rhizoctonia solani* than amphotericin B and hygromycin (Shentu et al. 2014a; Tijerino et al. 2011a). The mechanism and genes involved in the trichothecene biosynthesis in *Trichoderma* have been increasingly investigated. Although *Fusarium* and *Trichoderma* can synthesize trichothecenes, these species have remarkably different organizations of genes in the *tri* cluster and trichothecenes biosynthesis (Cardoza et al. 2011). *TRI* gene orthologues (*tri*) in *T. arundinaceum* and *T. brevicompactum* had been identified and characterized. The result showed that both *Trichoderma* species have a *tri* cluster with seven homologous genes in the *Fusarium TRI* cluster (Cardoza et al. 2011). Furthermore, the two *Trichoderma* species have the same organizations of genes in the *tri* cluster but different from that in *Fusarium* (Cardoza et al. 2011). Sequence and functional analyses demonstrated that the gene (*tri5*) responsible for the first step in trichothecene biosynthesis is located outside the cluster in both *Trichoderma* species but inside the cluster in *Fusarium* (Cardoza et al. 2011). Thus, analysis of the heterologous gene expression indicates that the two *T. arundinaceum* cluster genes (*tri4* and *tri11*) differ in function from their *Fusarium* orthologues (Cardoza et al. 2011). The *Tatri4*-encoded enzyme catalyzes only three of the four oxygenation reactions catalyzed by the orthologous enzyme in *Fusarium* (Cardoza et al. 2011). By contrast, the *Tatri4*-encoded enzyme in *T. arundinaceum* has the same function as that of *MrTri4*-encoded enzyme in *Myrothecium* (McCormick and Alexander 2007). The *Tatri11*-encoded enzyme catalyzes a reaction (trichothecene C-4 hydroxylation) that is completely different from that of the *Fusarium* orthologue (trichothecene C-15 hydroxylation) (Cardoza et al. 2011). However, the function of *tri3* gene in *T. arundinaceum* remains ambiguous. The lateral moiety at the C-4 position of the HA might be added to trichodermol through acetylation using an acyltransferase encoded by the *tri3* gene (Cardoza et al. 2011). The *tri5* gene has a significant role in the production of trichodermin in *T. brevicompactum* such that its overexpression increases trichodermin production and antimicrobial activity (Tijerino et al.2011a, b). However, whether *tri3* participates in the trichodermin biosynthesis remains unknown. The functions of *tri11* and *tri4* in *T. brevicompactum* should be verified. These genes are orthologues with *Tatri11* and *Tatri4* of *T. arundinaceum*.

We have identified and characterized the *tri* cluster of *T. brevicompactum* 0248, which can biosynthesize trichodermin (Yuan et al. 2016). The results indicated that *T. brevicompactum* 0248 has a 24,793 bp cluster that includes *tri14*, *tri12*, *tri11*, *tri10*, *tri3*, *tri4*, and *tri6* genes which are highly homologous to those in the *T. arundinaceum tri* cluster (Yuan et al. 2016). Furthermore, the *tri* cluster in strain 0248 is highly homologous to that of the reported strain *T. brevicompactum* IBT 40841. Strain 0248 and IBT 40841 clusters primarily differ in the size of the *tri11*–*tri12* intergenic region, which is 2287 bp in strain 0248 and 3000 bp in IBT 40841. In this study, we described the effects of the disruption of *tri11*, *tri3*, and *tri4* in *T. brevicompactum* 0248 on trichodermin production and gene expression of the other *tri* genes in trichodermin biosynthesis.

## Materials and methods

### Strains, culture media, and culture conditions

*Trichoderma brevicompactum* 0248 was isolated in our previous study and deposited in the China General Microbiological Culture Collection Center (CGMCC 6985) (Shentu et al. 2014b). The isolate was maintained on a potato-dextrose agar (PDA) slant medium at 4 °C until used.

*Agrobacterium tumefaciens* AGL-1, which was obtained from the Zhejiang Provincial Key Laboratory of Biometrology and Inspection and Quarantine, was grown in YEB medium with 100 µg/mL rifampicin at 28 °C before use for *A. tumefaciens*-mediated transformation (ATMT) (Lacorte et al. 1991). The *tri11*, *tri3*, and *tri4* genes in the *tri* cluster of *T. brevicompactum* 0248 were deleted by homologous recombination method as previously described (Brown et al. 2004; Kumar 2010).

The plasmid pSilent-1, which was provided by the Fungal Genetics Stock Center in USA, carried a hygromycin-resistant gene (*hph*) cassette. The plasmid pCAMBIA0380, which was obtained from the Zhejiang Provincial Key Laboratory of Biometrology and Inspection and Quarantine, was used for ATMT.

All primers were synthesized by Shanghai Sunny Biotechnology Co. Ltd and are listed in Additional file 1: Table S1, Additional file 2: Table S2, Additional file 3: Table S3.

### Construction of recombinant vector

**Fig. 1:**
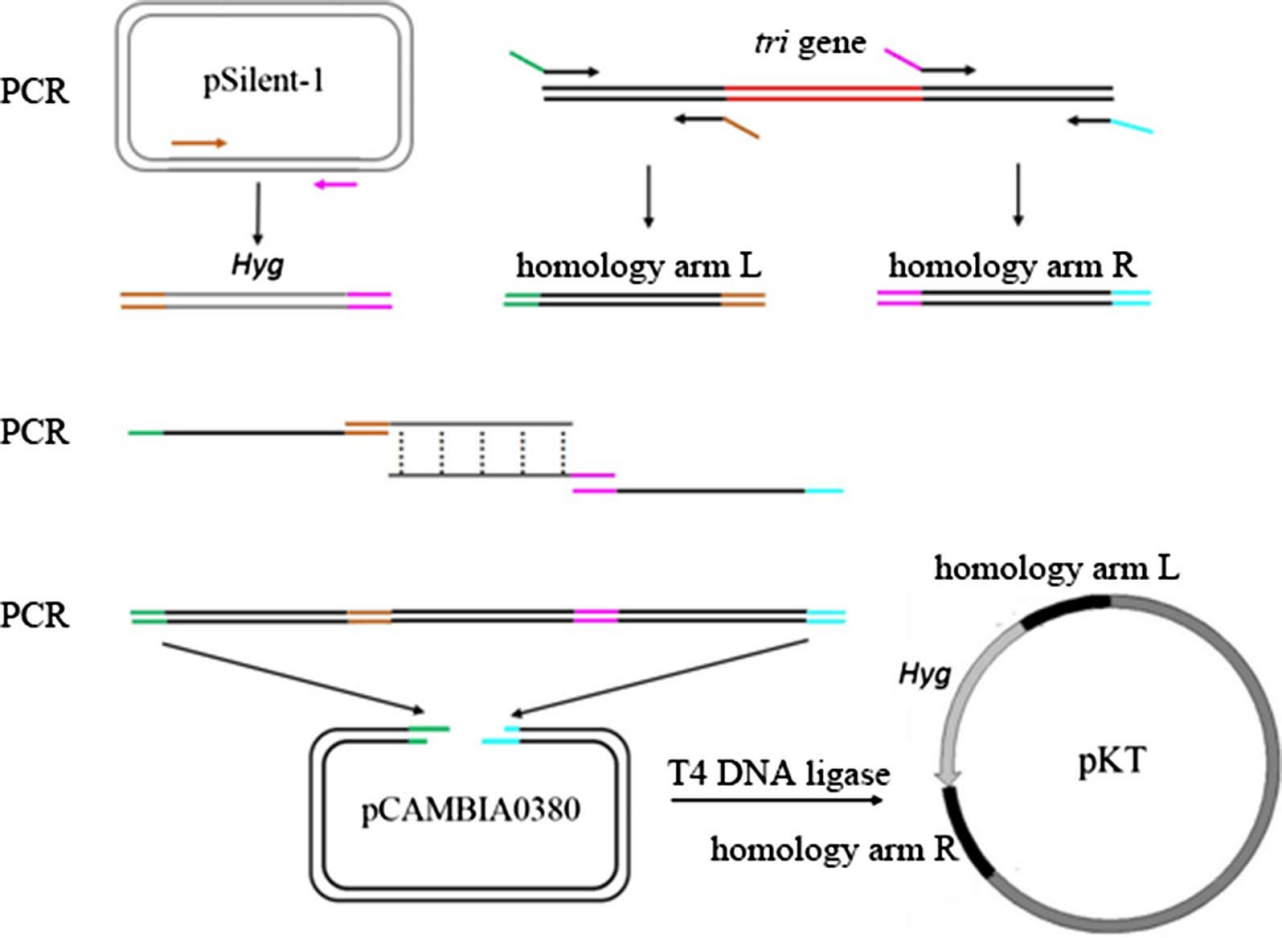
Construction of the recombinant plasmid for gene knockout. First, using plasmid pSilent-1 as the template, hygromycin resistance gene was amplified by PCR. An approximately 1-kb upstream DNA fragment before the *tri3, tri4*, and *tir11* gene start codon and another 1-kb upstream DNA fragment downstream of the *tri3*, *tri4*, and *tir11* gene stop codon were amplified to obtain homology L and R, respectively. Second, the above three linearized fragments were equimolarly mixed and cycled in a fusion PCR to generate a gene knockout fragment. Third, the corresponding gene knockout fragments were cloned into the *Bst*XI/*Xma*I sites of pCAMBIA0380 to generate the corresponding vectors designated as pKT3, pKT4, and pKT11

First, using plasmid pSilent-1 as template, *hph* was amplified by PCR with primers Ph-F/Ph-R (Additional file 1: Table S1) to obtain the *hph* expression cassette. Then, 1-kb upstream DNA fragment before the *tri11*, *tri3*, and *tir4* gene start codon was amplified using total DNA as template and corresponding primers P11-5F/P11-5R, P3-5F/P3-5R, and P4-5F/P4-5R. The forward primers P11-5F, P3-5F, and P4-5F contained the *Bst*XI site, while the reverse primers P11-5R, P3-5R, and P4-5R contained a 22 bp reverse complementary sequence of the *hph* cassette. Similarly, another DNA fragment downstream of the *tri11*, *tri3*, and *tir4* gene stop codon was amplified using primers P11-3F/P11-3R, P3-3F/P3-3R, and P4-3F/P4-3R, respectively. The forward primers P11-3F, P3-3F, and P4-3F contained a 22 bp overlapping sequence of the *hph* gene, while the reverse primers P11-3R, P3-3R, and P4-3R contained an *Xma*I site (Fig. 1). Then, the above three linearized fragments were equimolarly mixed and cycled in a fusion PCR to generate a gene knockout fragment (Cao et al. 2014). The corresponding gene knockout fragments were cloned into the *Bst*XI/*Xma*I sites of pCAMBIA0380 to generate the corresponding expression constructs designated as pKT11, pKT3, and pKT4 (Fig. 1).

### Transformation of *T. brevicompactum* 0248

The expression vectors pKT11, pKT3, and pKT4 were transformed using ATMT as described previously (Dos et al. 2004; Yang et al. 2011). The obtained transformants were confirmed by PCR and subcultured in PDA plates with 100 µg/mL hygromycin B for three generations to test the genetic stability.

### Quantitative RT-PCR (QRT-PCR) analysis

Total RNA of *T. brevicompactum* 0248 and Δ*tri11*, Δ*tri3*, and Δ*tri4* knockout strains were isolated using Spin Column Fungal Total RNA Purification Kit (Sangon, Shanghai, China). The total cDNA was obtained through reverse-transcription reaction using PrimeScript^®^ RT Reagent Kit with gDNA Eraser (TaKaRa, Dalian, China). QRT-PCR was performed in an Applied Biosystems StepOnePlus Real-Time PCR System using a SYBR^®^ Premix Ex TaqTM (TliRHaseH Plus) reagent (TaKaRa, Dalian, China) (Shentu et al. 2014a). The primers used in qRT-PCR are listed in Additional file 2: Table S2. The acceptable qRT-PCR standard curve (0.95 ≤ E ≤ 1.05, R^2^ ≥ 0.99) of the gene examined in this study was optimized by varying the annealing temperature and annealing time. The *β*-*tubulin* gene of *T. brevicompactum* 0248 was used as the reference gene (Shentu et al. 2014a). Quantification of the relative gene expression was analyzed by the 2^−ΔΔCt^ method.

### Analysis of trichodermin using gas chromatography (GC)

The fermentation broth was separated from the mycelia by filtration using a Buchner funnel and extracted exhaustively with ethyl acetate (v/v, 1:2). The organic fractions were combined and evaporated to dryness in a vacuum at 50 °C. The recovered residues were resuspended in methanol and analyzed using GC to quantify trichodermin (Shentu et al. 2008).

## Results

### Isolation of *tri11*, *tri3*, and *tri4* deletion mutants

**Fig. 2:**
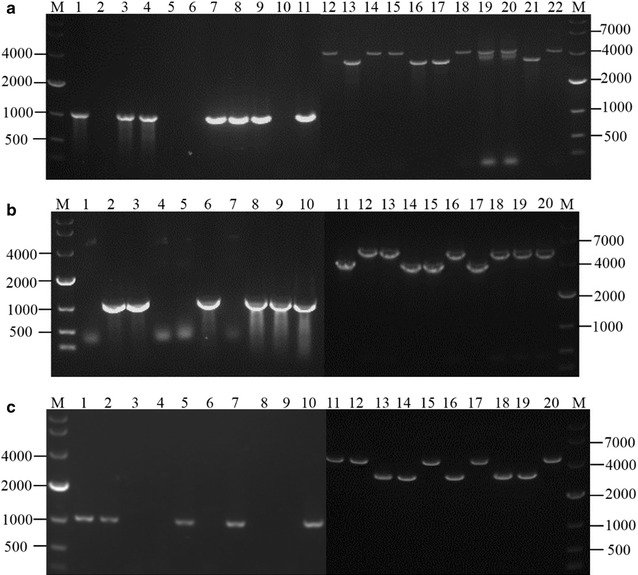
Agar gel analysis. **a** Verification of *tri3* deletion. Numbers from 1 to 10 and 12 to 21 refer to the arbitrarily chosen transformants. Numbers 11 and 22 refer to the wild strain. The numbers and their corresponding transformants are as follows: 1 and 12, Δ*tri3*-1; 2 and 13, Δ*tri3*-2; 3 and 14, Δ*tri3*-3; 4 and 15, Δ*tri3*-4; 5 and 16, Δ*tri3*-5; 6 and 17, Δ*tri3*-6; 7 and 18, Δ*tri3*-7; 8 and 19, Δ*tri3*-8; 9 and 20, Δ*tri3*-9; and 10 and 21, Δ*tri3*-10. Lanes 2, 5, 6, and 10 did not show the expected band of 1.1 kb. This result indicated that *tri 3* was deleted in Δ*tri3*-2, Δ*tri3*-5, Δ*tri3*-6, and Δ*tri3*-10, respectively. Furthermore, Δ*tri3*-2, Δ*tri3*-5, Δ*tri3*-6, and Δ*tri3*-10 (lanes 13, 16, 17, and 21, respectively) showed the expected band of 3.6 kb (reduced by approximately 0.7 kb). The ORF of the *tri3* was replaced successfully by the hygromycin cassette in these mutants. **b** Verification of *tri11* deletion. Numbers from 1 to 9 and 11 to 19 refer to the arbitrarily chosen transformants, while numbers 10 and 20 refer to the wild strain. The numbers and their corresponding transformants are as follows: 1 and 11, Δ*tri11*-1; 2 and 12, Δ*tri11*-2; 3 and 13, Δ*tri11*-3; 4 and 14, Δ*tri11*-4; 5 and 15, Δ*tri11*-5; 6 and 16, Δ*tri11*-6; 7 and 17, Δ*tri11*-7; 8 and 18, Δ*tri11*-8; and 9 and 19, Δ*tri11*-9. Lanes 1, 4, 5, and 7 did not show the expected band of 1.1 kb. This result indicated that *tri11* was deleted in Δ*tri11*-1, Δ*tri11*-4, Δ*tri11*-5, and Δ*tri11*-7, respectively. Furthermore, Δ*tri11*-1, Δ*tri11*-4, Δ*tri11*-5, and Δ*tri11*-7 (lanes 11, 14, 15, and 17, respectively) showed the expected band of 3.6 kb (reduced by approximately 0.7 kb). The ORF of the *tri11* was replaced successfully by the hygromycin cassette in these mutants. **c** Verification of *tri4* deletion. Numbers from 1 to 9 and 11 to 19 refer to the arbitrarily chosen transformants, while numbers 10 and 20 refer to the wild strain. The numbers and their corresponding mutants are as follows: 1 and 11, Δ*tri4*-1; 2 and 12, Δ*tri4*-2; 3 and 13, Δ*tri4*-3; 4 and 14, Δ*tri4*-4; 5 and 15, Δ*tri4*-5; 6 and 16, Δ*tri4*-6; 7 and 17, Δ*tri4*-7; 8 and 18, Δ*tri4*-8; and 9 and 19, Δ*tri4*-9. Lanes 3, 4, 6, 8, and 9 did not show the expected band of 1.1 kb. This result indicated that *tri4* was deleted in Δ*tri4*-3, Δ*tri4*-4, Δ*tri4*-6, Δ*tri4*-8, and Δ*tri4*-9, respectively. Furthermore, Δ*tri4*-3, Δ*tri4*-4, Δ*tri4*-6, Δ*tri4*-8, and Δ*tri4*-9 (lanes 13, 14, 16, 18, and 19, respectively) showed the expected band of 3.6 kb (reduced by approximately 0.7 kb). The ORF of the *tri4* was replaced successfully by the hygromycin cassette in these mutants

Using the gene-deletion plasmid and ATMT, transformants were selected on PDA plates with 100 µg/mL hygromycin B. Then, these transformants were further identified by PCR using the primers P11N-R/P11N-F, P3N-R/P3N-F, and P4N-R/P4N-F (Additional file 3: Table S3) to verify *Δtri11*, *Δtri3*, and *Δtri4 mutant*s, respectively. Figure 2a shows that 10 transformants were arbitrarily chosen for PCR analysis, of which transformants 2, 5, 6, and 10 (lanes 2, 5, 6, and 10, respectively) did not exhibit the expected 1.1 kb band. Furthermore, primer pair P3W-R/P3W-F was designed according to the flanking sequence of the upstream/downstream DNA fragments of *tri3.* This pair was used to verify whether transformants 2, 5, 6, and 10 had the expected 3.6 kb band. The length of the *hph* cassette and ORF of the *tri3* were 1.1 and 1.78 kb, respectively. The band of lane 22 from the fragment of the wild strain was 4.3 kb. If the ORF of the *tri3* was replaced successfully by the *hph* cassette in mutants, transformants 2, 5, 6, and 10 (lanes 13, 16, 17, and 21, respectively) should show the expected 3.6 kb band (reduced by approximately 0.7 kb). These expected results were observed (Fig. 2a), indicating that *Δtri3* mutants were obtained successfully. *Δtri11* and *Δtri4* mutants were verified with the same method (Figs. 2b and c). Four transformants (lanes 1, 4, 5, and 7) in which the *hph* cassette replaced the ORF of the *tri11* gene were identified (Fig. 2b). Five transformants (lanes 3, 4, 6, 8, and 9) in which the *hph* cassette replaced the ORF of the *tri4* gene were also determined (Fig. 2c).

### Effect of *tri3* deletion on the expression of the *tri* genes

**Fig. 3:**
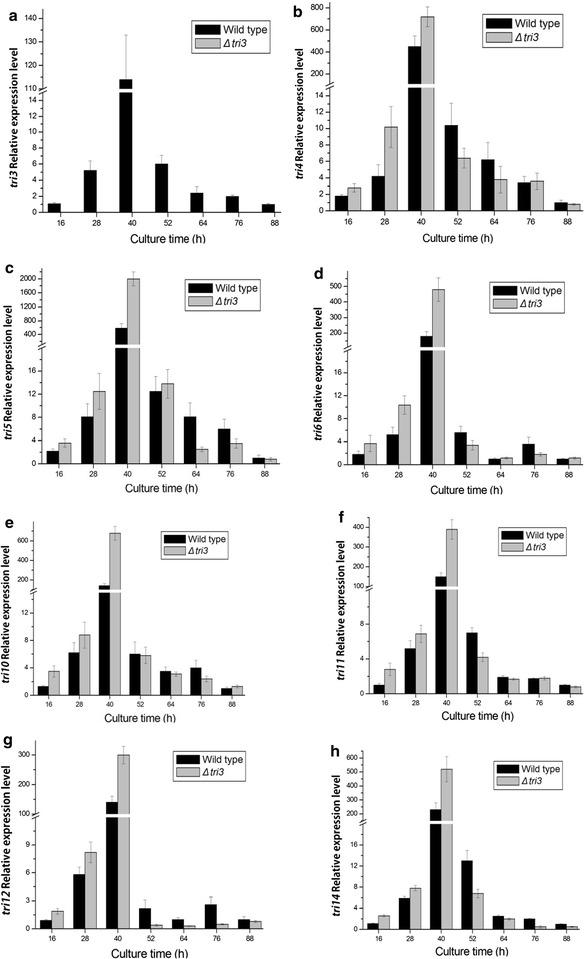
Expression of *tri* genes in Δ*tri3* mutant and wild strain. The quantification of *tri3* gene expression in the different culture times was analyzed by 2^−ΔΔCt^ method. The wild strain cultured for 88 h was used as control and *β*-*tubulin* as reference gene. **a** *tri3*, **b** *tri4*, **c** *tri5*, **d** *tri6*, **e** *tri10*, **f**-*tri11*, **g** *tri12*, and **h** *tri14*

The relative expression levels of the *tri* genes in the *Δtri3* mutant were analyzed and compared with those of the wild-strain 0248. The expression of the *tri3* gene in the wild strain changed with culture time (16, 28, 40, 52, 64, 76, and 88 h), with the highest expression level observed at fermentation for 40 h (Fig. 3a). The *tri3* expression was not detected in the *Δtri3* mutant at each time point. Surprisingly, the deletion of *tri3* resulted in the significant upregulation of the expression of *tri4*, *tri5*, *tri6*, *tri10*, *tri11*, *tri12*, and *tri14* genes compared with that of the wild type after 40 h of culture. The expression of these genes in *Δtri3* mutant had remarkably declined from 52 to 88 h (Fig. 3).

### Effect of *tri4* deletion on the expression of the *tri* genes

**Fig. 4:**
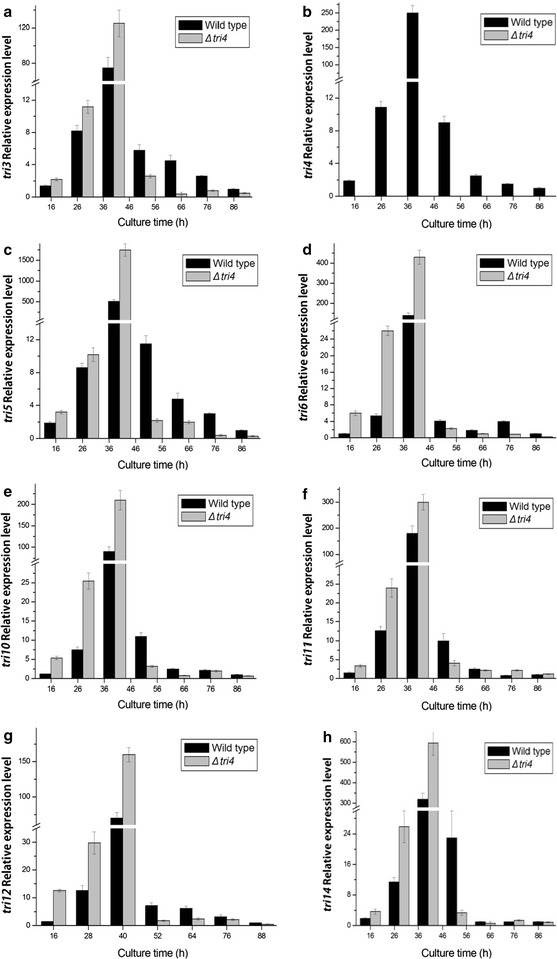
Expression of *tri* genes in Δ*tri4* mutant and wild strain. The quantification of *tri4* gene expression in the different culture times was analyzed by 2^−ΔΔCt^ method. The wild strain cultured for 88 h was used as control and *β*-*tubulin* as reference gene. **a** *tri3*, **b** *tri4*, **c** *tri5*, **d** *tri6*, **e** *tri10*, **f** *tri11*, **g** *tri12*, and **h** *tri14*

We determined whether the deletion of *tri4* gene affected the transcription of *tri* genes. The relative expression levels of *tri3*, *tri4*, *tri5*, *tri6*, *tri10*, *tri11*, *tri12*, and *tri14* in the *Δtri4* mutant and wild strain 0248 were detected. The *tri4* expression was not detected in the *Δtri4* mutant at each time point (Fig. 4). The deletion of *tri4* positively affected the expression of *tri3*, *tri5*, *tri6*, *tri10*, *tri11*, *tri12*, and *tri14* compared with the wild-type strain at 40 h. After 52 h of fermentation, the expression levels of these genes declined.

### Effect of *tri11* deletion on expression of the *tri* genes

**Fig. 5:**
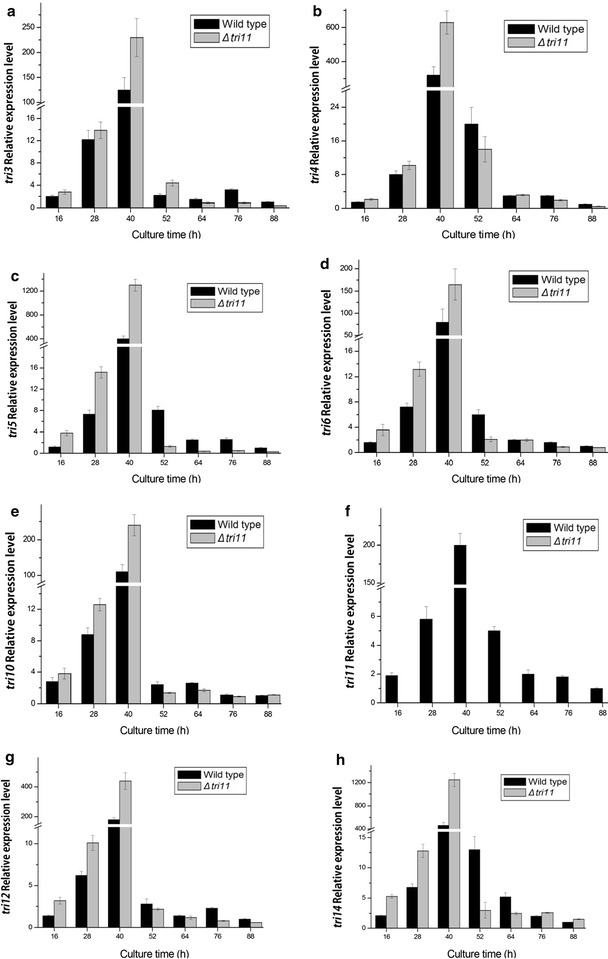
Expression of *tri* genes in Δ*tri11* mutant and wild strain. The quantification of *tri11* gene expression in the different culture times was analyzed by 2^−ΔΔCt^ method. The wild strain cultured for 88 h was used as control and *β*-*tubulin* as reference gene. **a** *tri3*, **b** *tri4*, **c** *tri5*, **d** *tri6*, **e** *tri10*, **f** *tri11*, **g** *tri12*, and **h** *tri14*

The *tri11* deletion process was successful, and no expression of *tri11* was found in the *Δtri11* mutant. After 52 h of culture, the expression level of *tri3* was higher in the *Δtri11* mutant than in the wild strain. At other time points, relative expression levels of *tri3*, *tri4*, *tri5*, *tri6*, *tri10*, *tri12*, and *tri14* in the *Δtri11* mutant were higher than in the wild strain (Fig. 5).

### Effect of *tri3*, *tri4*, or *tri11* deletion on trichodermin production

**Fig. 6:**
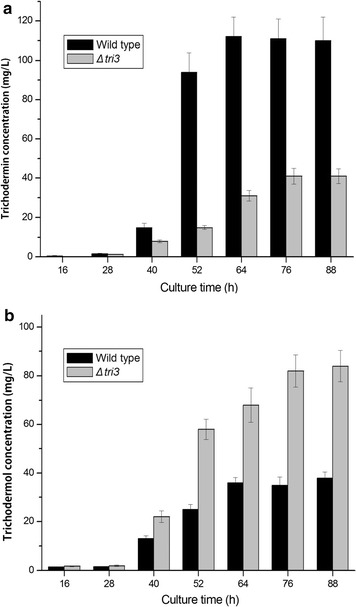
**a** Trichodermin production by *Δtri3* mutants and wild strain 0248. **b** Trichodermol production by *Δtri3* mutants and wild strain 0248

Given that trichodermin is the sole product of the *tri* cluster in *T. brevicompactum* 0248, the concentrations of trichodermin in the culture extracts were analyzed by GC in both wild strain and *Δtri* gene mutants. No trichodermin was detected in the *Δtri4* and *Δtri11* mutants. That is, *tri4* or *tri11* deletion directly affected trichodermin biosynthesis by *T. brevicompactum*. However, *Δtri3* mutant could still biosynthesize trichodermin, although *tri3* gene was deleted (Fig. 6a). Prolonged cultivation time resulted in increased trichodermin concentration in the *Δtri3* mutant and strain 0248, although the trichodermin yield by *Δtri3* mutant was lower than that of the wild strain at 76 h of fermentation by approximately 40 mg/L trichodermin. Deletion of *tri3* gene resulted in reduced trichodermin content. Furthermore, high abundance of trichodermol in the *Δtri3* strain was detected after 40 h cultivation, contrary to the quite low trichodermol content in the wild-type culture extracts (Fig. 6b). It was shown that trichodermol was accumulated in the culture broth of *Δtri3* mutant.

## Discussion

*Trichoderma* is an interesting fungus because of its important application in biocontrol (Malmierca et al. 2012). However, *T. brevicompactum* has not been well-studied among *Trichoderma*, because *T. brevicompactum* (IBT 9471) had been erroneously considered as *T. harzianum* (ATCC 90237) because of substantial shared micromorphology of these two species (Nielsen et al. 2005; Degenkolb et al. 2008). Until 2005, this IBT 9471 strain has been reclassified as *T. brevicompactum* on the basis of phylogenetic lineage within the morphological species *T. brevicompactum* and trichothecene production (Nielsen et al. 2005; Shentu et al. 2014a). Previous studies reported that *T. brevicompactum* is one of the *Trichoderma* species that produces trichodermin, which exhibits strong antifungal activity and has high biotechnological value (Tijerino et al. 2011a, b). Therefore, conducting systematic studies on this species is necessary and urgent.

In this study, *tri4* and *tri11* deletion mutants were generated to evaluate the roles of *tri4* and *tri11* in trichodermin biosynthesis. *Tatri4* catalyzes the addition of oxygen at C-2, C-12, and C-11, and this process converts trichodiene to isotrichodiol in *T. arundinaceum*. Deletion mutant strain *Δtri4* did not produce trichodermin in strain 0248, and this phenomenon was previously observed for *T. arundinaceum Δtri4* mutants (Cardoza et al. 2011; Malmierca et al. 2012). This result suggested that *tri4* is essential for trichodermin biosynthesis in *T. brevicompactum*. *Tatri11* controls the hydroxylation at C-4, and this process converts 12,13-epoxytrichothec-9-ene (EPT) to trichodermol in *T. arundinaceum*. Deletion mutant strain *Δtri11* also did not biosynthesize trichodermin in *T. brevicompactum* 0248. Thus, these results first proved the necessity of *tri11* for trichodermin biosynthesis in *T. brevicompactum*.

In *T. arundinaceum*, Tri3 is hypothesized to acetylate oxygen at C-4 of the trichothecene skeleton based on the amino acid sequence and motif, HXXXDG, which is indicative of acetyl-transferases (Murray and Shaw 1997). However, the function of *tri3* in *Trichoderma* has not been verified yet. In our study, *Δtri3* deletion mutant strain was obtained. The trichodermin in *Δtri3* cultures was detected using GC, and the results showed lower concentration in the *Δtri3* deletion mutant than in the wild type. As expected, the *tri3* gene was involved in the biosynthesis of trichodermin by *T. brevicompactum*. Furthermore, high abundance of trichodermol in the *Δtri3* strain was detected, contrary to the quite low trichodermol content in the wild-type culture extracts. These results demonstrated that deletion of *tri3* gene in *T. brevicompactum* 0248 reduced the trichodermin content and accumulated trichodermol. Thus, *tri3* gene was responsible for the acetyl moiety to the C-4 oxygen in *T. brevicompactum*.

Small amount of trichodermin was still detected in the *Δtri3* mutants. This phenomenon was also observed for deletion mutants of *Fusarium sporotrichioides* (McCormick et al. 1996; Garvey et al. 2009). Trace amounts of trichothecene T-2 toxin were detected in *tri3* mutants of *F. sporotrichioides* (McCormick et al. 1996). The small amount of trichodermin suggested the presence of another acetyl-transferase that can convert trichodermol into trichodermin but with lower efficiency. This result implied that an unidentified enzyme analogous to the *tri3* acetylase from *T. brevicompactum* strain was probably responsible for the catalysis of the C-4 hydroxyl group of trichodermol. Many unigenes homologous to the acyltransferase-encoding genes were found in our previous transcriptome analysis of *T. brevicompactum* 0248 (Shentu et al. 2014a). In summary, our results indicated that *tri3* gene was responsible for the last step of the acetylation of the C-4 oxygen in *T. brevicompactum*.

Interestingly, QRT-PCR analyses showed that before 52 h cultivation, the loss of function of *tri3*, *tri4*, or *tri11* led to the upregulation of the *tri* genes. These results were in agreement with the feedback regulation mechanism. To our knowledge, the end-product and intermediate metabolites play a significant role in the regulation of metabolic pathways by directly or indirectly regulating the genes involved in metabolic pathways (Goelzer et al. 2008; Herrgard et al. 2006). Previous studies showed that the synthesis of trichodermin was mainly concentrated during the incubation period of 40–60 h (Yuan et al. 2016). Therefore, the lower yield of trichodermin in *Δtri3* mutant or intermediate metabolites in *Δtri4* and *Δtri11* mutants may serve as a signal to trigger the feedback regulation. Thus, the expression of *tri* genes was upregulated, and synthesis of enzymes was induced.

In conclusion, *T. brevicompactum* is an important species because of its high biocontrol potential. The biosynthesis of trichothecenes by *T. brevicompactum* has been elucidated. It is first proved that *tri4* and *tri11* are essential for trichodermin biosynthesis by *T. brevicompactum*. This study is also the first to report the function of *tri3* in *Trichoderma*, and the results confirmed the previous hypothesis on the *tri3* function in the biosynthesis of trichothecenes.

## Additional files

**Additional file 1: Table S1.** Primers used to construct plasmid pKT.

**Additional file 2: Table S2.** Primers used for qRT-PCR.

**Additional file 3: Table S3.** Primers used to verify transformants.

## Abbreviations

PDA: potato-dextrose agar
GC: gas chromatography
ATMT: *Agrobacterium tumefaciens*-mediated transformation
