# Supplementary material for: Tri11, tri3, and tri4 genes are required for trichodermin biosynthesis of Trichoderma brevicompactum
Source: AMB Express. 2018 Apr 17;8:58. doi: 10.1186/s13568-018-0585-4 (PMC5904096; doi:10.1186/s13568-018-0585-4)
Supplement: Supplementary file 1 — Additional file 1: Table S1. Primers used to construct plasmid pKT. [file 13568_2018_585_MOESM1_ESM.doc]

Table S1 Primers used for the construction of plasmid pKT

| Primer name | Primer sequence(**5’-3’**) |
| --- | --- |
| Ph-F | GATATGAAAAAGCCTGAACT |
| Ph-R | ACTCTATTCCTTTGCCCTCG |
| P11-5F | TAGGCCACCATGTTGGGCCCTGGAGTGTTGTACTTCGTAT |
| P11-5R | AGTTCAGGCTTTTTCATATCTATCCGTTCAGTCCCTTTGGGAC |
| P11-3F | CGAGGGCAAAGGAATAGAGTGACGAATTGGAACCTAGTGT |
| P11-3R | GTGGACTCCTCTTAAAGCTTCAGTAATGATAGGTGAGCAC |
| P3-5F | TAGGCCACCATGTTGGGCCCCAGTCAGAAGCATAAAACGAG |
| P3-5R | AGTTCAGGCTTTTTCATATCTATGGCGCTCAATTAAAATGTG |
| P3-3F | CGAGGGCAAAGGAATAGAGTATGTATGATGCGTTGGGAGTG |
| P4-5F | TAGGCCACCATGTTGGGCCCCCATATCGGTACAAAACACAC |
| P4-5R | AGTTCAGGCTTTTTCATATCGATTGATGAACCACTAGCACG |
| P4-3F | CGAGGGCAAAGGAATAGAGTGTTATGATTTATGGCACTCG |
| P4-3R | GTGGACTCCTCTTAAAGCTTGATTAATACGGAGTAATCCAC |
