# Supplementary material for: Tri11, tri3, and tri4 genes are required for trichodermin biosynthesis of Trichoderma brevicompactum
Source: AMB Express. 2018 Apr 17;8:58. doi: 10.1186/s13568-018-0585-4 (PMC5904096; doi:10.1186/s13568-018-0585-4)
Supplement: Supplementary file 2 — Additional file 2: Table S2. Primers used for qRT-PCR. [file 13568_2018_585_MOESM2_ESM.doc]

Table S2 Primers used for qRT−PCR

| Gene name | Primer sequence(**5’-3’**) |
| --- | --- |
| *tri14*F | AGGAATGAATCCCAAAT |
| *tri14*R | GACATCCGAGACGAAA |
| *tri12*F | TGGCAACTGGCGATGGA |
| *tri12*R | GGCAAAAGAGGGAGGATG |
| *tri11*F | CTGATTGGAAAGCAGAGC |
| *tri11*R | AGCCCGAGATAGGAAGGA |
| *tri10*F | TTTGGCAGCACGCAGAT |
| *tri10*R | GCGATGGCAATAGAAGG |
| *tri3*F | TTCCTGCGAGCATCACC |
| *tri3*R | TTGCCTCCTCCCGACTG |
| *tri4*F | TTCGGTCCCATTAGTC |
| *tri4*R | CCATCGCTCAGGTTTA |
| *tri6*F | GTCCCGCCATTGACATCT |
| *tri6*R | CGCCATTATCGCCAACAC |
| *tri5*F | TTCTAAATGCCCGACCAC |
| *tri5*R | GAGCCACGGAAACCCT |
| *β-tubulin* F | ATGCCACCCTGTCTATGC |
| *β-tubulin* R | CGAAGGTCGGAGTTGAGC |
