# Supplementary material for: Tri11, tri3, and tri4 genes are required for trichodermin biosynthesis of Trichoderma brevicompactum
Source: AMB Express. 2018 Apr 17;8:58. doi: 10.1186/s13568-018-0585-4 (PMC5904096; doi:10.1186/s13568-018-0585-4)
Supplement: Supplementary file 3 — Additional file 3: Table S3. Primers used to verify transformants. [file 13568_2018_585_MOESM3_ESM.doc]

Table S3 Primers used for verification of transformants

| Primer name | Primer sequence(**5’-3’**) |
| --- | --- |
| P11N-F | GGTTGCACTGTTTCACGACG |
| P11N-R | GACCACTGTGCGAAAGCGAC |
| P11W-F | GTACCTTGAGCATCGACTC |
| P11W-R | GTCCCATCTTGAGTGCCAGA |
| P3N-R | CCTGAGGATGACGAAGAGG |
| P3N-F | GAATGGCATTGGATTCCTAG |
| P3W-R | GCTTAGCGACAACACCTTAC |
| P3W-F | CGAATGGATCGGCTAGAGTC |
| P4N-R | CCTCATCTTAGATAAATCAC |
| P4N-F | GAATGGTATGCTCCTTGTAC |
| P4W-R | CGGTGGAATACTCTGTAGAG |
| P4W-F | TGAATGACCAAGGTACTCAC |
